# Subsidies for Pregnant Women with “Genuinely Unavoidable Special Reasons”

**DOI:** 10.31662/jmaj.2021-0211

**Published:** 2022-02-28

**Authors:** Shunji Suzuki

**Affiliations:** 1 Department of Obstetrics and Gynecology, Japanese Red Cross Katsushika Maternity Hospital, Tokyo, Japan

**Keywords:** public subsidies for delivery, the revised Hospitalization Assistance Policy Program, genuinely unavoidable special reasons, Japan

## Abstract

Here, we present two cases of women with social problems whose delivery and hospitalization costs were fully subsidized by the revised Hospitalization Assistance Policy Program. The presence of the program is required to be enlightened and expanded in Japan.

Based on the Child Welfare Act, in Japan, delivery costs for pregnant women with serious economic problems are subsidized through the Hospitalization Assistance Policy Program ^(1)^. The system allows affected women to give birth at specified obstetric institutions. In August 2019, the Japanese Ministry of Health, Labor and Welfare expanded the scope of the subsidies for delivery in women with social and/or economic problems (https://ajhc.or.jp/siryo/20190808.pdf) because in Japan, there have been some cases where pregnant women with the problems could not receive the subsidies for delivery because of lack of approach from the staff of the local governments (Table 1) ^(2)^. For example, if an obstetrics medical institute discovers that there are pregnant women who receive no livelihood support from their partners to escape from the intimate partner violence (IPV), their deliveries are supported by the public subsidy program. In addition, the delivery costs have also been subsidized when there is “a genuinely unavoidable special reason in pregnant women,” which is at the discretion of each local government.

**Table 1.** Differences in the Reasons for Subsidizing Delivery Costs in the Hospitalization Assistance Policy Program before and after August 2019.

Period	Reason for receiving subsidies
Until July 2019	1. Pregnant women receiving the livelihood protection
	2. Households exempted from the residence tax
	3. Households in which the income tax is <¥8,400 (about 100 US dollars) per year
From August 2019	1-3 above
	4. Specified expectant mothers certified by the local government as unable to pay the delivery cost

Here, we present here two cases subsidized by the revised program.

Case 1: A 32-year-old primiparous woman at 34 weeks of gestation was referred to our institute for obstetric management through the Maternal and Child Health Division of a local government, located more than 30 km away from our hospital. The woman was hidden and protected in a maternal and child life support facility (domestic violence shelter for abuse victims run by the government), which was kept secret from her partner and his acquaintances. Although we were informed of her identity, she used her pseudonym for the chart displays in the institute. She developed hypertensive disorders and gave birth to a healthy male neonate by vacuum extraction at 38 weeks of gestation. The delivery and hospitalization costs (approximately $ 5,000) were subsidized in full. After the delivery, the Maternal and Child Division of the closed local government contacted the Division in a distant town where her relatives lived, and at 1 month after the delivery, she and her infant moved to the place together.

Case 2: A 26-year-old primiparous woman, who did not have enough money, visited our institute at 33 weeks of gestation because she was unable to return to home country due to the COVID-19 pandemic. Our medical social workers consulted with the Welfare Division of the local government with her “truly unavoidable special reason” and her hospitalization and delivery costs were publicly subsidized. She was admitted to the hospital on the premise of induction of delivery at 35 weeks of gestation because of hypertensive disorders with fetal growth restriction. She gave birth to a healthy female neonate by Cesarean section at 36 weeks of gestation. Following delivery, she stayed near the institute under the protection of the Maternal and Child Health Division of the local government until they could return to their home country.

Japan may have been considered a wealthy country; however, the poverty of young people is now especially one of major social problems. At our institute, one of main perinatal centers in Tokyo, approximately 100 women give birth under the subsidies of the Hospitalization Assistance Policy Program every year ^(1)^; however, there were 2-5 women with “genuinely unavoidable special reasons” not eligible for the subsidies every year. Although the local government official had arranged for the subsidy as much as possible, their delivery costs were sometimes paid in installments over many years under interest-free conditions or were borne by our institute. In the cases, most of the delivery costs were exempted based on the philosophy of our institute contributing to the local community by medical care, health, and welfare. However, those women were occasionally missing and/or were out of reach. In addition, this inflicted financial and mental burden for our institute.

In Japan, pregnant women at high risk and in need of extra support after giving birth have been recognized as “specified expectant mothers” to take special support measures ^(3)^. To support mothers and their children at present and in the future, obstetricians, pediatricians, clinical psychologists, midwives, and medical social workers in our institute have developed a multi-professional collaboration, also cooperating with the local government (Figure 1) ^(4)^. Because appropriate and individual supports will be required, it is of great importance that the Japanese Ministry of Health, Labor and Welfare has gradually relaxed the standards in supporting women with truly unavoidable special reasons in the last few years ^(2)^.

**Figure 1.**
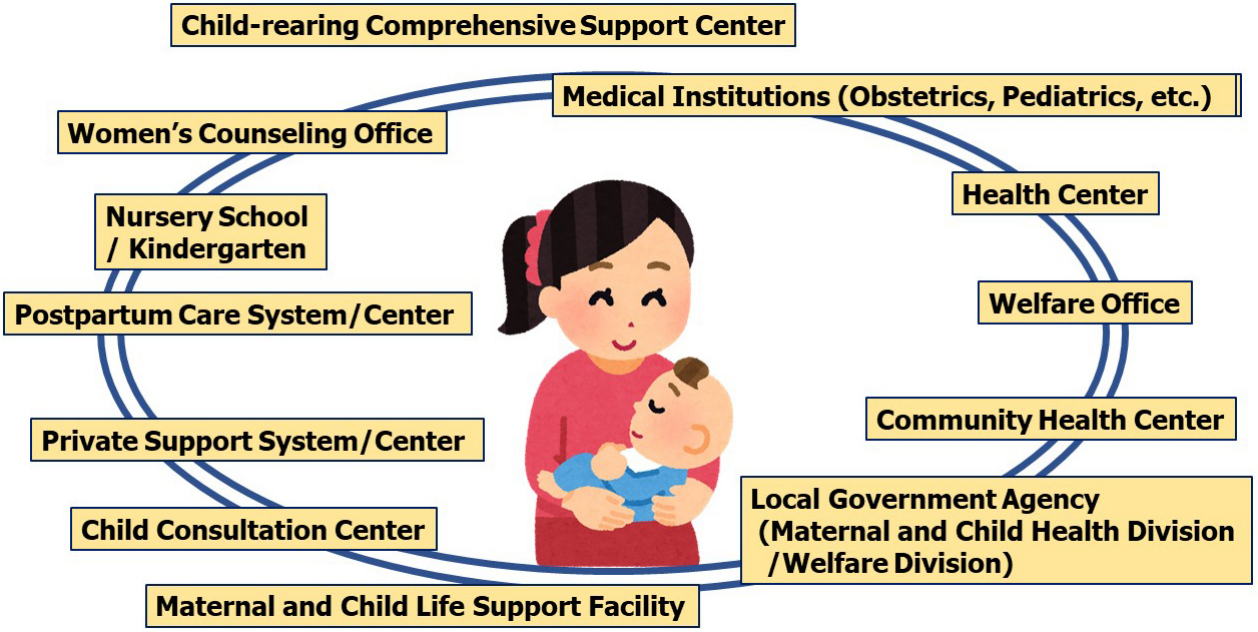
Illustration of multi-professional collaboration with local governments in an obstetric medical institute.

We are afraid that some pregnant women may have never visited obstetrics medical institutes because they were unfamiliar with the revised program. We are also concerned that some medical institutions may not be aware of the mentioned revision. For this reason, these subsidies should be widely promoted in Japan.

## Article Information

### Conflicts of Interest

None

### Author Contributions

Shunji Suzuki has designed the study and collected the data. In addition, he accepts the responsibility for the entire content of this manuscript and approves of its submission.

### Approval by Institutional Review Board (IRB)

The study protocol was approved by the Ethics Committee of Japanese Red Cross Katsushika Maternity Hospital (2021-15). From all subjects, informed consent concerning retrospective analyses was obtained. The data supporting the results of the current study are available in the article.
